# UDP-sugar pyrophosphorylase is essential for arabinose and xylose recycling, and is required during vegetative and reproductive growth in Arabidopsis

**DOI:** 10.1111/tpj.12116

**Published:** 2013-02-02

**Authors:** Claudia Geserick, Raimund Tenhaken

**Affiliations:** Department Cell Biology, Plant Physiology, University of SalzburgHellbrunnerstr. 34, Salzburg, 5020, Austria

**Keywords:** nucleotide sugar, salvage pathway, cell wall precursor, UDP-arabinose, metabolism

## Abstract

Numerous nucleotide sugars are needed in plants to synthesize cell wall polymers and glycoproteins. The de novo synthesis of nucleotide sugars is of major importance. During growth, however, some polymers are broken down to monosaccharides. Reactivation of these sugars into nucleotide sugars occurs in two steps: first, by a substrate-specific sugar-1-kinase and, second, by UDP-sugar-pyrophosphorylase (USP), which has broad substrate specificity. A knock-out of the *USP* gene results in non-fertile pollen. By using various genetic complementation approaches we obtained a strong (>95%) knock-down line in *USP* that allowed us to investigate the physiological role of the enzyme during the life cycle. Mutant plants show an arabinose reduction in the cell wall, and accumulate mainly two sugars, arabinose and xylose, in the cytoplasm. The arabinogalactanproteins in *usp* mutants show no significant reduction in size. USP is also part of the myo-inositol oxygenation pathway to UDP-glucuronic acid; however, free glucuronic acid does not accumulate in cells, suggesting alternative conversion pathways of this monosaccharide. The knock-down plants are mostly sterile because of the improper formation of anthers and pollen sacks.

## Introduction

**Figure 1:**
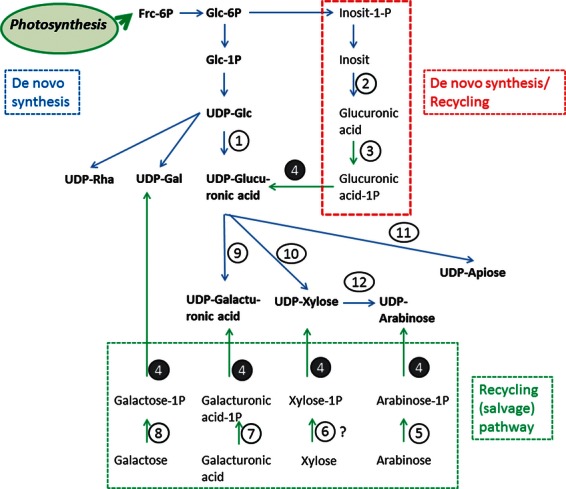
Partial overview of the nucleotide sugar pathway in plants, showing the main reaction in which USP is involved. The de novo pathway uses photosynthesis products to make UDP-GlcA, an important precursor for other UDP sugars of pectins and hemicelluloses. In a side pathway, inositol is oxygenated to GlcA, and thereafter reactivated to UDP-GlcA, involving enzymes of the sugar production and recycling pathways. The lower part of the scheme shows the classical recycling enzymes, which usually consist of a substrate-specific sugar-1-kinase and a broad range substrate using USP: 1, UDP-glucose dehydrogenase; 2, myo-inositol oxygenase; 3, glucuronokinase; 4, UDP-sugar pyrophosphorylase (USP); 5, arabinokinase; 6, putative xylokinase; 7, galacturonokinase; 8, galactokinase; 9, UDP-glucuronic acid-4-epimerase; 10, UDP-xylose synthase; 11, UDP-apiose xylose synthase; 12, UDP-xylose-epimerase.

Nucleotide sugars provide the building blocks for plant cell wall polymers and glycoproteins. These glycosyl donors are generated initially through de novo pathways, which provide the different UDP or GDP sugars by interconversion from UDP-Glc or GDP-Man (Seifert, 2004; Reiter, 2008; Bar-Peled and O'Neill, 2011). During plant growth some of the cell wall polymers or sugar chains from glycoproteins are turned over and degraded to monomers. To be able to reuse these sugars, a parallel salvage pathway network is present in plants (Feingold and Avigad, 1980). Sugars are phosphorylated at the C1 position by specific kinases, and are subsequently converted into the corresponding UDP sugars: for example, by UDP-sugar-pyrophosphorylase (USP). This enzyme was purified from *Pisum sativum* (pea) sprouts, and peptide sequence data led to the isolation of the corresponding gene (Kotake *et al*., 2004). USP is highly conserved in plants, but was recently also identified in the genome of human parasites like *Trypanosoma* (Yang and Bar-Peled, 2010) and other protozoans. The USP enzyme from pea converts several sugar-1-phosphates into the corresponding UDP sugars, a property that is retained in the enzymes from Arabidopsis (Litterer *et al*., 2006; Kotake *et al*., 2007) or *Trypanosoma* (Yang and Bar-Peled, 2010). Because of the broad substrate specificity, USP is sometimes also referred to as Sloppy. The typical substrates of USP are shown in Figure 1. A T–DNA insertion disrupting the *USP* gene of Arabidopsis was shown to result in improper pollen development (Schnurr *et al*., 2006). A homozygous T-DNA mutant could not be obtained, as the male gametophyte does not transmit the defect *usp* allele. Heterozygous *usp*/*USP* plants showed no visible phenotypes, but had a 50% lower USP enzyme activity compared with the wild type. The function of USP for the metabolism of plants is unknown. USP has the highest affinity to UDP-glucuronic acid-1-phosphate (UDP-GlcA-1-P), a substrate of the myo-inositol oxygenase (MIOX) pathway to UDP-GlcA (Figure 1). This may suggest that the major function of USP is a role as terminal enzyme of the MIOX pathway to UDP-GlcA; however, a strong knock-down in a quadruple mutant of the four MIOX genes shows no visible differences compared with the wild type, and is fertile (Endres and Tenhaken, 2011). Based on microarray data and the promoter::GUS fusion (Kotake *et al*., 2007), USP is widely expressed during the plant life cycle, with a slight preference for flowers. This argues against a pollen-specific role. The Arabidopsis *mur4* mutant has a defect in UDP-xylose-4-epimerase, which provides UDP-arabinose for polymer synthases in the Golgi apparatus (Burget and Reiter, 1999). One phenotype of *mur4* is a reduction in length of the glycosylation of arabinogalactan proteins. Feeding arabinose to *mur4* mutants restores the wild-type glycosylation pattern of arabinogalactan proteins, a pathway involving arabinokinase (Dolezal and Cobbett, 1991) and USP.

We were interested to learn more about the role of USP for plant metabolism, and performed a series of genetic complementation experiments of heterozygous *usp*/*USP*, and searched for homozygous mutants in transformed complementing lines. This allowed us to test the importance of different possible pathways of USP.

## Results

**Figure 2:**
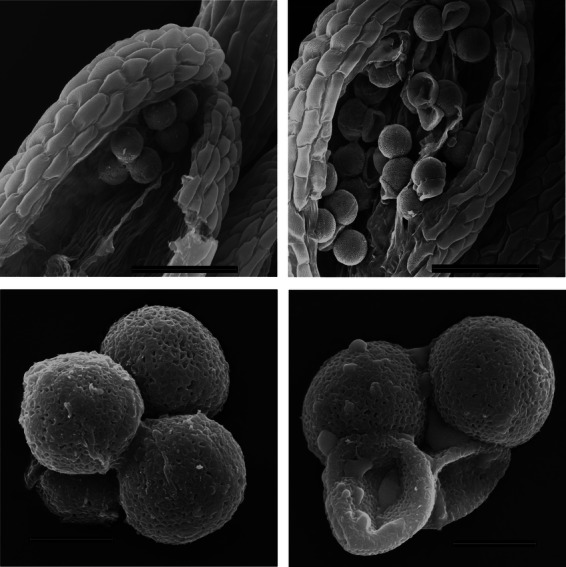
Anthers from the wild type (left) and heterozygous *usp* mutants (right) were analyzed by scanning electron microscopy. Scale bars: 50 μm for the anthers (upper row); 10 μm for the single pollen tetrad (lower row).

We self-fertilized two independent heterozygous T-DNA insertion lines in the *USP* gene and genotyped the descendants in order to obtain a homozygous knock-out line for *usp*. Consistent with the report from Schnurr *et al*. (2006), we were unable to find any homozygous *usp* mutants among the more than 100 plants tested. We performed scanning electron microscopy of anthers and pollen tetrads to analyse the defect in *usp* mutants (Figure 2). Whereas all of the pollen in wild-type plants develops normally and shows filled pollen grains, the tetrads in *usp* always show two filled and two collapsed pollen grains. The Mendelian inheritance during meiosis is consistent with a single gene defect in *usp*. In order to find out why the *usp* knock-out mutation is lethal, we tried to complement heterozygous *usp* plants with different DNA constructs, and searched in the transformed lines for homozygous *usp* descendants. USP uses many sugar-1-phosphates (glucose, galactose, glucuronic acid, xylose, arabinose and galacturonic acid) as substrates to convert them into the corresponding UDP sugars, as shown in Figure 1 (Litterer *et al*., 2006; Kotake *et al*., 2007). The approach with different complementation constructs allowed us to test several hypotheses about the function of USP, in particular which of the substrates of USP is likely to be responsible for the lethal pollen phenotype. The different constructs used for this approach are summarized in Table 1. We first tested whether UDP-GlcA biosynthesis is limited in *usp* plants causing the abortion of pollen development. During most of the life cycle, plants synthesize most of their UDP-GlcA by the oxidation of UDP-Glc by the enzyme UDP-glucose dehydrogenase (Sharples and Fry, 2007; Endres and Tenhaken, 2011; Reboul *et al*., 2011). In pollen however, an alternative pathway is more important, in which myo-inositol is first oxygenated to GlcA, which is subsequently phosphorylated by glucuronokinase to GlcA-1-P and finally converted into UDP-GlcA by USP (Figure 1). It has been shown previously by (Kroh and Loewus, 1968) that this pathway provides most of the precursors for pectins in *Lilium* pollen tubes. The enzyme UDP-glucose dehydrogenase was expressed in heterozygous kd-usp mutants either under the control of the ubiquitin10 promoter or in a dexamethasone inducible vector system to increase the pool of UDP-GlcA. We thereby tested, whether too low concentrations of UDP-GlcA are responsible for the lethal pollen phenotype in usp-mutants. We genotyped ∼100 descendants from two independent transformants for each construct, but failed to identify a homozygous *usp* mutant among them.

**Table 1:** Overview of the complementation constructs used to transform heterozygous *usp* mutants

Promotor	Structural gene	Viable homozygous T-DNA insertion lines obtained
*pMiox4*	*AtUSP::GFP*	No
*pLat52*	*AtUSP::GFP*	No
*pUbq10*	*AtUSP::GFP*	**Yes**
Dex-inducible promotor	*GmUDP-Glc DH*	No
*pMiox4*	*GmUDP-Glc DH::GFP*	No
*pLat52*	*GmUDP-Glc DH::GFP*	No
*pUbq10*	*GmUDP-Glc DH::GFP*	No
*pCaMV35S*	*GMUDP-Glc DH::GFP*	No
*pUbq10*	*AtUDP-Glc-pyrophosphorylase*	No
*pUbq10*	*AtUDP-Glc-pyrophosphorylase::GFP*	No

Siblings of primary transformants were screened by PCR for a homozygous *usp* mutation. The promotors used in these experiments were either the anther/pollen-specific *pMIOX4* or *pLat52* promotors or the constitutive *pUbq10* or *CaMV35S* promotors. The structural genes were: Arabidopsis USP (*AtUSP*) fused to GFP, the UDP-glucose dehydrogenase from soybean (*GmUDP-Glc DH*) or the UDP-glucose pyrophosphorylase from Arabidopsis. Typically approximately 100 descendants from at least two independent transformants were genotyped. No indicates that no homozygous usp-mutants was obtained among the 100 tested descendants.

In a second approach we tested the hypothesis that the recycling of glucose and the formation of UDP-glucose is critical during pollen maturation. The rationale behind this experiment refers to studies by Eschrich (1966), in which a massive callose breakdown was described during pollen maturation. We therefore expressed UDP-glucose pyrophosphorylase (Kleczkowski *et al*., 2004) under the control of the pUbq10 promoter in heterozygous *usp* lines. This approach also failed to obtain homozygous *usp* knock-out lines among the siblings.

**Figure 3:**
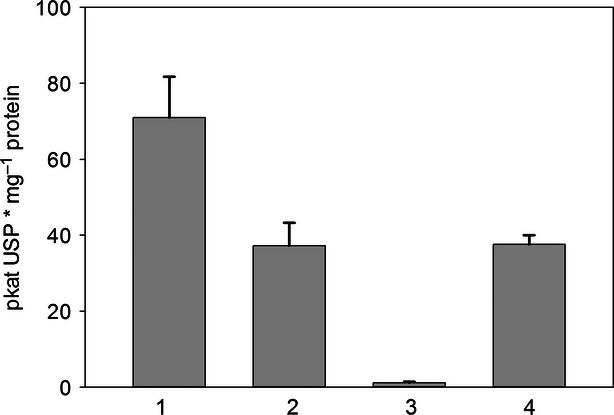
USP enzyme activity. The enzyme activity of USP was measured from leaf extracts using glucuronic acid-1-phosphate as a substrate. Products were separated and quantified by HPLC: 1, wild type; 2, heterozygous *usp*/*USP*; 3, *kd-usp*; 4, homozygous *usp* mutant, but complemented with *pUSP::USP*.

Next we tried to complement the heterozygous *usp* lines with a *USP::GFP* fusion construct under the control of different promoters. The transgenic lines using the anther- and pollen-specific MIOX4 promoter (Kanter *et al*., 2005) failed to produce homozygous *usp* siblings. Finally, we used the ubiquitin10 promoter to drive the *USP::GFP* fusion. Transformed plants did not show a clearly visible GFP fluorescence, suggesting a low protein abundance of the USP::GFP fusion. Nevertheless, we obtained homozygous *usp* mutants from several independent primary transformants, but at a low frequency (in a ratio of roughly 1:20, instead of the theoretical ratio of 1:3). These homozygous *usp* mutants are comparable with knock-down plants, as they express USP::GFP fusion protein at a low level. The enzyme activity of USP was therefore measured using GlcA-1-P as a substrate. This substrate was chosen because USP has the highest affinity to it, no other side reactions are known and because the product of the reaction UDP-GlcA can easily be detected on HPLC. As shown in Figure 3, heterozygous *usp* lines have ∼50% activity compared with wild-type USP. The complemented *usp/usp USP::GFP* plants have a very low but detectable USP activity, which is ∼3% of the wild-type level, confirming the status as a strong knock-down mutant (*kd-usp*).

**Figure 4:**
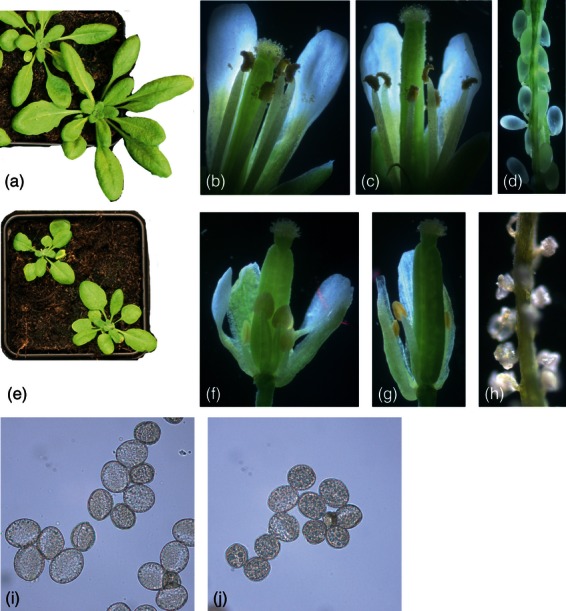
Phenotype of the *kd-usp* mutant: (a) rosette of a 4-week old wild-type plant grown under short-day conditions; (b) young wild-type flower; (c) mature wild-type flower; (d) opened developing wild-type silique; (e) rosette of a 4-week-old *kd-usp* plant grown under short-day conditions; (f) young *kd-usp* flower; (g) mature *kd-usp* flower; (h) opened developing *kd-usp* silique; (i) pollen from wild-type plants; (j) pollen from *kd-usp* plants.

The phenotype of *kd-usp* plants is shown in Figure 4. Rosette leaves of *kd-usp* remain smaller compared with wild-type plants. The mutants develop normal organ structures, except that the flowers fail to produce (viable) seeds. Seeds were obtained with a very low frequency, below 0.1% of the wild-type yield, making it almost impossible to collect seeds for extensive experiments. The flowers of *kd-usp* have shorter anthers than the wild type and remain wet and closed, with the pollen sticking together. We also tried to manually fertilize flowers of *kd-usp* by streaking anthers with pollen sacs across the stigma in order to overcome any problem that might be caused by the shorter anthers. This approach was not successful, most likely because the pollen sacs did not release the pollen properly.

**Figure 5:**
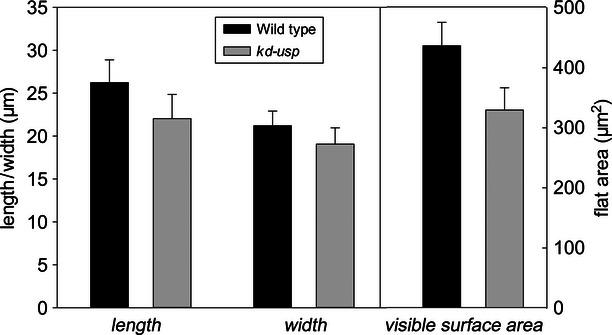
Pollen diameters. Pollen from wild-type plants and *kd-usp* mutants were spread on a microscopic slide and photographed; the length and width was determined using ImageJ.

The pollen from wild-type and *kd-usp* mutants differ in size (Figure 5). The average size of wild-type pollen is 26.2 μm in length and 21.2 μm in width, whereas the pollen from *kd-usp* plants are smaller, with a length of 22 μm and a width of 19.1 μm. The flat surface area of the pollen is 437 μm^2^ in the wild type and 330 μm^2^ in *kd-usp*.

**Figure 6:**
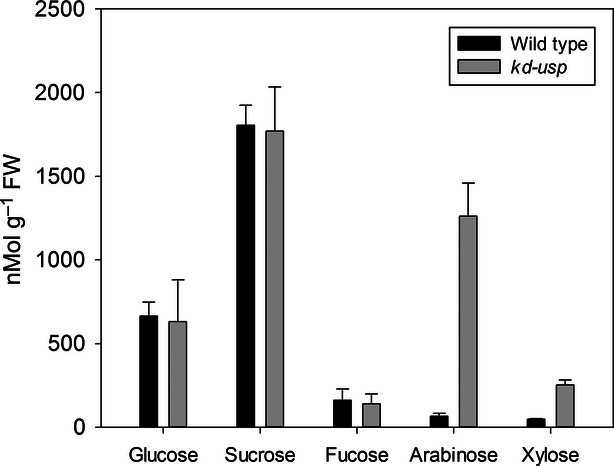
Sugar metabolites. Extracts from 4-week-old leaves were separated on HPLC and soluble sugars were determined by pulsed amperometric detection. Data are shown for the wild type and *kd-usp* mutants (*n* = 4).

The *kd-usp* phenotype during vegetative growth already suggests an important function of USP for normal plant development beyond the requirement in pollen growth. We also compared gene expression data for *USP* and other enzymes related to nucleotide sugar recycling pathways from microarray studies in an organ-specific manner (Figure S1). The *USP* gene is expressed in all plant organs, with a preference for pollen. The sugar-1-kinases, acting in concert with USP, are also expressed during the life cycle of the plant, pointing to nucleotide sugar recycling not only in pollen but in the whole plant. As USP accepts many sugar-1-phosphates as substrates, we were interested to find out which of them are of major importance for normal metabolism. We anticipated that sugars and sugar-1-phosphates should accumulate in the cell if the salvage pathway to UDP sugars is blocked in *kd-usp* plants. Indeed, we found the accumulation of two sugars, arabinose and xylose, in metabolite extracts of *kd-usp*. Arabinose-1-phosphate and xylose-1-phosphate could not be measured directly: the electrochemical detection has a low sensitivity and reference compounds are not commercially available. All other sugars remain at the same level in wild-type and mutant plants (Figure 6).

**Figure 7:**
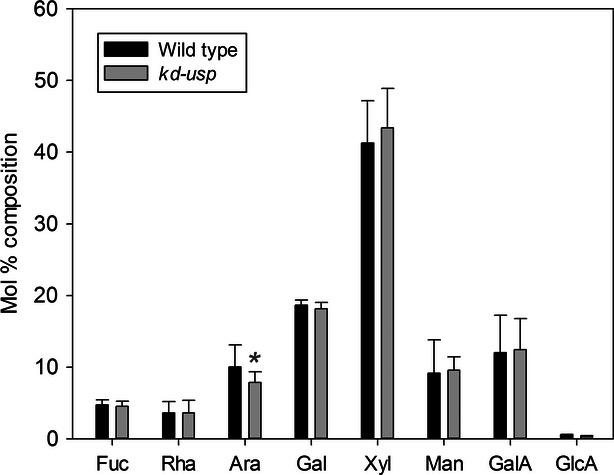
Cell wall sugar composition. Cell walls were hydrolyzed in trifluoroacetic acid (TFA), and liberated sugars were separated on HPLC and detected by pulsed amperometric detection. Data are shown for wild-type plants and *kd-usp* mutants (*n* = 8); *P < 0.1.

Next we compared the cell wall composition and found only a small, but significant, reduction in arabinose in *kd-usp*, suggesting that in wild-type plants part of the arabinose from polymers is liberated during development and recycled to UDP-arabinose via USP (Figure 7). For xylose we did not see statistically significant differences in the cell wall composition of wild type and *kd-usp* plants, although xylose clearly accumulates in *kd-usp* mutants. Interestingly, GlcA as an intermediate of the MIOX pathway to UDP-GlcA does not accumulate in *kd-usp* mutants.

The *mur4* mutant has a lower arabinose content, which results in shorter sugar chains of arabinogalactanproteins (AGPs; Burget and Reiter, 1999). We therefore isolated total AGPs from the wild type, an *ara1-1* mutant with a defect in arabinokinase (Dolezal and Cobbett, 1991) and *kd-usp* plants, and size-separated the glycoproteins on an agarose gel (Figure S2). The average size of AGPs is, however, similar in wild type, *ara1-1* and *kd-usp* mutants.

## Discussion

The recycling of sugars into nucleotide sugars usually requires two steps: a phosphorylation at the C1 position, which is catalyzed by sugar-specific kinases, and USP, which takes at least five different sugar-1-phosphates as substrates, and converts them into the corresponding UDP sugars. The most important substrates for USP *in vivo* are therefore unknown. USP is encoded by a single-copy gene in Arabidopsis, and a knock-out in the *USP* gene is lethal, as pollen carrying a *usp* allele do not develop normally (Schnurr *et al*., 2006), which we confirmed in our initial analysis.

Here, we show that a strong knock-down mutant with <5% residual activity is compromised in vegetative growth. This suggests that the recycling of nucleotide sugars occurs during the vegetative growth phase, and that blocking this recycling inhibits the normal growth of, for example, rosette leaves. Thus, the function of USP is not limited to metabolism in pollen development, but is presumably necessary throughout the life cycle of the plant. This is also backed up by gene expression data for enzymes involved in nucleotide sugar recycling, which are usually expressed in all plant organs, although often with a pollen preference (Figure S1).

The observation that USP can convert many different sugar-1-phosphates into their UDP sugars does not necessarily imply an equal importance of the recycling of all sugars. Pollen tubes are fast-growing cells with a high concentration of pectins in their cell wall. The direct pectin precursor is UDP-galacturonic acid, which is derived from UDP-GlcA by epimerization (Gu and Bar-Peled, 2004). It has been shown previously by Kanter *et al*. (2005) and Kroh and Loewus (1968) that the myo-inositol pathway, which requires the USP as the final enzyme to produce UDP-GlcA, is highly active in pollen. Microarray data reveal a high expression of the genes for myo-inositol oxygenase, glucuronokinase and USP (Figure S1). Thus, the knock-out of *USP* would interrupt the MIOX pathway to UDP-GlcA, and could severely reduce pectin precursor availability; however, a strong knock-down quadruple mutant in all four MIOX genes shows no visible phenotype changes, but an upregulation of the genes for UDP-glucose dehydrogenase, which probably compensates for a reduction in the metabolite UDP-GlcA caused by *MIOX* downregulation (Endres and Tenhaken, 2011). Our approach to overexpress UDP-glucose dehydrogenase in *kd-usp* mutants to increase the level of UDP-GlcA failed to complement the *usp* mutation, suggesting that the cause of pollen lethality in *kd-usp* mutants is not the non-functional MIOX pathway to UDP-GlcA, but a defect in the recycling of other sugars.

Another function of nucleotide sugar recycling pathways in pollen could be the usage of pistil exudates, as suggested by Labarca and Loewus (1972). This role of USP may well be important for the better growth of pollen tubes, but cannot explain the lethal pollen phenotype that we observed in *kd-usp* mutants. The pollen in *usp* do not develop properly (Figure 2), and must therefore have a problem in early maturation. Schnurr *et al*. (2006) localized the defect in *usp* to a missing intine layer. The possible nutrition function of USP would come into play far later, when germinated pollen grows through the style.

In *Oryza sativa* (rice), pollen development requires an active UDP-Glc pyrophosphorylase, as co-suppressed plants develop their pollen normally until meiosis, but fail to deposit callose shortly afterwards (Chen *et al*., 2007), leading to male-sterile rice plants. We tried a complementation with the Arabidopsis UDP-Glc pyrophosphorylase, but failed to obtain homozygous *usp* plants, suggesting that this function is not the critical step in *usp* mutants.

Unexpectedly we did not find an accumulation of GlcA in the cell, indicating that alternative pathways for GlcA usage exist. Most likely GlcA is converted to ascorbic acid along an animal-like pathway (Linster and Van Schaftingen, 2007), which at least is functional in feeding studies (Davey *et al*., 1999) in Arabidopsis cell cultures, and acts independently from the regular pathway via GDP-mannose.

We found an accumulation of arabinose and xylose in *kd-usp* lines. For technical reasons we could not measure arabinose-1-phosphate and xylose-1-phosphate, which will most likely also accumulate in the cells. Nevertheless, this allows several conclusions about the function of USP in plants. First of all, arabinose and xylose are substrates for the salvage pathway via USP *in vivo*, and are most likely derived from the turnover of cell wall polymers. As we are working with knock-down lines, the accumulation of arabinose and xylose would be far higher in a true knock-out line. The cell wall composition of *kd-usp* plants show a small but significant reduction in arabinose, suggesting that the recycling of arabinose contributes to the cellular UDP-arabinose pool.

### Is the reduction of arabinose causing the *kd-usp* phenotype?

Work from the Reiter laboratory (Burget *et al*., 2003) has identified UDP-xylose-4-epimerase in plants, the enzyme that contributes most to the pool of UDP-arabinose in the novel biosynthesis pathway. The *mur4-1* mutant with a defect in UDP-xylose-4-epimerase has strongly reduced levels of arabinose in the cell wall, but shows almost no visible phenotype compared with wild-type plants. Therefore the reduction in UDP-arabinose alone is unlikely to be responsible for the phenotype of *kd-usp* mutants. One important difference between *mur4-1* and *kd-usp*, however, is the cellular compartment, in which UDP-arabinose is generated by the respective pathway. UDP-xylose-4-epimerase is located in the Golgi apparatus, and thus mainly contributes to the Golgi pool of UDP-arabinose used to synthesize arabinans, petic side chains and arabinogalactan proteins. The enzymes for the salvage pathway, arabinokinase (Dolezal and Cobbett, 1991) and USP are localized in the cytoplasm, and thus contribute to a cytosolic pool of UDP-arabinose. Putative targets in the cytoplasm requiring UDP-arabinose are glycosyltransferases for secondary compounds (Yonekura-Sakakibara *et al*., 2008), or unknown enzymes synthesizing soluble heteroglycans, with an arabinose, galactose and xylose core, involved in starch metabolism (Fettke *et al*., 2005).

The mutant *ara1-1*, which has a defect in the cytoplasmic enzyme arabinokinase, dies during seedling development when low mM concentrations of arabinose are applied to mutant plants (Dolezal and Cobbett, 1991). This might suggest a problem for plants when arabinose accumulates in the cytoplasm. Whether the accumulation of arabinose in *kd-usp* mutants is responsible for the phenotype remains to be shown.

Free galactose was shown to inhibit growth in plants (Ordin and Bonner, 1957), suggesting that the removal of free sugars is critical for plant growth. The role of USP is then to convert the intermediate sugar-1-phosphates into nucleotide sugars as precursors for novel polysaccharides. Whether galactose or galactose-1-phosphate is toxic remains to be shown, but a recent paper by Egert *et al*. (2012) suggests that it is not the free galactose, but galactose-1-phosphate or a misbalance in the sugar-1-phosphate and nucleotide-sugar network, that causes the growth defects.

Many mutations in Arabidopsis cause a stop in pollen development. Recently, Levitin *et al*. (2008) identified two arabinogalactan proteins (AGP6 and AGP11) that are essential for pollen growth. We analyzed AGPs to compare the average size of total arabinogalactan chains and found no difference to wild-type plants; however, *mur4-1* mutants clearly show shorter sugar chains of AGPs on average (Burget and Reiter, 1999). Therefore, it is rather unlikely that a modification of AGPs is causing the phenotype of *kd-usp* mutants.

Finally, the accumulation of xylose in *kd-usp* is surprising, as it suggests a so far unknown recycling pathway for xylose via xylose-1-phosphate. Based on feeding radioactive precursor to *Lilium* pollen, it was assumed until now that xylose recycling occurs via xylulose and the pentose phosphate pathway into fructose/glucose, which would not require USP activity (Rosenfield and Loewus, 1978; Rosenfield *et al*., 1978). Nucleotide sugar pathways vary between pollen and leaves. A conversion of xylose into glucose via the pentose phosphate pathway in pollen does not allow a generalization for the whole plant. We are not aware of any publication in which the existence of a xylose kinase was excluded. In a recent review, xylose to xylose-1-phosphate conversion was described as an unresolved pathway (Bar-Peled and O'Neill, 2011). From the accumulation of xylose in *kd-usp* mutants we conclude that a xylosekinase exists in plants, but remains to be identified in future work. Consistent with this, Kotake *et al*. (2004) and Litterer *et al*. (2006) have already shown that xylose-1-phosphate is a substrate of USP.

## Experimental procedures

### Plant material

Wild-type *Arabidopsis thaliana* plants (Col), and two SALK T-DNA mutant lines (SALK_092647; SALK_030169)(Alonso *et al*., 2003) were obtained from the Arabidopsis stock center (NASC, http://arabidopsis.info). Plants were grown in standard soil (Einheitserde ED73) in a growth chamber at 22°C with ∼130 μm photons m^–2^ s^–1^ and a 10-h (16-h) light period for short (long) day plants.

### PCR

DNA from Arabidopsis leaves was extracted by the standard cetyl trimethylammonium bromide (CTAB)-buffer method. Genotyping of T-DNA mutants was performed by PCR with primers USP-WT-Salk_030169F and USP-WT-Salk_030169R, or with USP-WT-Salk_092647F and USP-WT-Salk_092647R for wild-type alleles. The T-DNA was detected with primers Salk_030169-T-DNA and Salk-LB or Salk_092647-T-DNA and SalkLB using the following conditions: 94°C for 3 min, followed by 32 cycles of 95°C for 10 s, 59°C for 20 s and 72°C for 1 min. Primer sequences are listed in Table S1.

### Constructs for complementation

All PCR primers used to generate constructs are listed in Table S1. The gateway-compatible plant expression vector pMDC84 (Curtis and Grossniklaus, 2003) was used to construct complementation vectors for the *usp* mutant. The *CaMV35S* promotor was removed by restriction with *HinD*III and *Spe*I, and replaced by either the *pMiox4*-(1480 bp), *pLAT52*- (1540 bp) or *pUbiquitin10*-(1934 bp) promotor flanked by *HinD*III and *Spe*I restriction sites. The promoters were amplified from genomic Arabidopsis DNA by PCR using the primers pMiox4-F and pMiox4-R, pLAT52-F and pLAT52-R, pUbq10-F and pUbq10-R, and Phusion (Thermo Scientific, http://www.thermoscientific.com) proof-reading polymerase under conditions recommended by the manufacturer.

The *USP* gene was amplified from a first-strand cDNA from Arabidopsis RNA with Phusion DNA-polymerase using primers attB1-AtUSP and attB2-AtUSP-with-stop or attB2-AtUSP-without-stop to allow Gateway recombination later. The gene for UDP-glucose dehydrogenase from *Glycine max* (soybean; Tenhaken and Thulke, 1996) was amplified with primers attB1-UDP-GlcDH and attB2-UDP-GlcDH-with-stop or attB2-UDP-GlcDH-without-stop. The gene for Arabidopsis UDP-Glc pyrophosphorylase (At5 g17310) was amplified with primers attB1-UGPase and attB2-UGPase-with-stop or attB2-UGPase-without-stop.

Clones without terminal stop codons were used for translational fusions with GFP in pMDC84. The attB1 and attB2 sites were extended in a second PCR reaction with primers attB1-adaptor and attB2-adaptor to give full-length attB1 and attB2 sites.

Constructs were verified by restriction mapping and sequencing, and then transformed into *Agrobacterium* (GV3101 pMP90). Colonies from *Agrobacterium* were tested for the plasmid by colony-PCR, and used for transformation of heterozygous SALK_030169 and SALK_092647 plants using the floral-dip standard method. Seeds were collected and selected for transformants on half-strength MS-phytagel plates with 5 g l^–1^ sucrose and 30 μg ml^–1^ hygromycin. Primary transformants were transferred to soil and genotyped for the presence of the complementation construct, the SALK_030169 (092647) T-DNA and the absence of the USP wild-type allele. Typically more than 40 siblings were analyzed for each construct.

Plants with the construct containing the dexamethasone-inducible gene for UDP-glucose dehydrogenase from soybean were sprayed with 20 μm dexamethasone every day during flowering.

### USP enzyme assay

Leaves from 4-week-old rosettes, grown under short-day conditions, were extracted in the cold with buffer [50 mm Tris/Cl, pH 7.8, 2 mm MgCl_2_, 10% glycerol, 0.5 mm phenylmethylsulfonyl fluoride (PMSF)]. After centrifugation (13 000 *g* for 5 min at 4°C), the supernatant was desalted in the same buffer using a NAP5 size-exclusion column (GE Healthcare, http://www.gehealthcare.com). USP activity was measured in the forward reaction using glucuronic acid-1-phosphate as substrate. The substrate was synthesized during the assay by the addition of recombinant glucuronokinase from Arabidopsis. The USP assay (200 μl in total) consisted of 50 mm Tris/Cl, pH 8, 2 mm MgCl_2_, 50 μl of desalted leaf extract, 1 mm ATP, 1 mm UTP, 1 mm glucuronic acid and 0.01 units of recombinant glucuronkinase (Pieslinger *et al*., 2010). The assay was performed at 30°C for 20 min and stopped by heat inactivation (95°C for 3 min). A 1:3 dilution of the assay was applied to a Partisil SAX10 HPLC-column. Products were separated by a phosphate buffer gradient: buffer A, 10 mM Na-phosphate, pH 2.8; buffer B, 750 mm Na-phosphate, pH 3.7. The HPLC-program used was *t*_0_ 2% B, *t*_13_ 9% B, *t*_22_ 9% B, *t*_27_ 36% B and *t*_50_ 60% B. The flow rate was 0.5 ml min^–1^. The product UDP-GlcA eluted around 30 min and was quantified at 262 nm using authentic standards (Sigma-Aldrich, http://www.sigmaaldrich.com) as a reference.

### Cell wall and metabolite analysis

Cell walls were prepared from 4-week-old plant leaves. Material was homogenized in liquid N_2_, extracted with different solvents, acid hydrolyzed and finally separated on HPLC, as described by Reboul *et al*. (2011). Metabolites from leaves (40–60 mg fresh weight) were extracted from liquid N_2_ homogenized material in 250 μl methanol/chloroform (7:3) for 1 h at 4°C. Sugars were recovered by the addition of 300 μl H_2_O and a 10-min extraction with shaking. The upper phase was dried in an Eppendorf vacuum concentrator to dryness and redissolved in 200 μl H_2_O for HPLC analysis. The quantities of metabolites were compared with authentic sugar standards.

### AGP analysis

Total AGP proteins were extracted from homogenized leaves according to the method described by Burget and Reiter (1999). Samples were separated by electrophoresis on a 1% agarose gel (5 V cm^–1^ for 2 h) and stained for several hours in 15 μm β-Glc-Yariv. Samples were slightly destained in 1% NaCl solution and photographed.

## SUPPORTING INFORMATION

**Figure S1.** Heat map of expression data in different plant organs for genes involved in nucleotide sugar biosynthesis.

**Figure S2.** Yariv staining of gel-separated arabinogalactan proteins from wild type, ara1-1 and kd-usp mutant plants.
