# Supplementary material for: UDP-sugar pyrophosphorylase is essential for arabinose and xylose recycling, and is required during vegetative and reproductive growth in Arabidopsis
Source: Plant J. 2013 Feb 2;74(2):239–47. doi: 10.1111/tpj.12116 (PMC3659416; doi:10.1111/tpj.12116)

Supplement

Supplement Table S1

Primer	Sequence
attB1-USP	AAAAAGCAGGCTTCATGGCTTCTACGGTTGATTC
attB2-AtUSP-with-stop	GAAAGCTGGGTTCAATCTTCAACAGAAAATTTGCC
attB2-AtUSP-without-stop	GAAAGCTGGGTATCTTCAACAGAAAATTTGCCA
attB1-UDP-GlcDH	AAAAAGCAGGCTTCATGGTGAAGATTTGCTGCAT
attB2-UDP-GlcDH-with-stop	GAAAGCTGGGTTATGCCACAGCAGGCAT
attB2-UDP-GlcDH-without-stop	GAAAGCTGGGTTGCCACAGCAGGCATGT
attB1-UGPase	AAAAAGCAGGCTTCATGCGTTATGCGGTATATTCTTT
attB2-UGPase-with-stop	GAAAGCTGGGTTCACAGATCCTCTGGACCGT
attB2-UGPase-without-stop	GAAAGCTGGGTCAGATCCTCTGGACCGTTGA
attB1-adaptor	GGGGACAAGTTTGTACAAAAAAGCAGGCTTC
attB2-adaptor	GGGGACCACTTTGTACAAGAAAGCTGGGT
Salk_092647-T-DNA	TGAGCAAGTGTGGATTTCTACTA
Salk_030169-T-DNA	ATTGTCTTCCATTTCGTGATCTG
USP-WT-Salk_030169F	ATTGTCTTCCATTTCGTGATCTG
USP-WT-Salk_030169R	TGAGCAAGTGTGGATTTCTACTA
USP-WT-Salk_092647F	GCTACAGGTAAACCGATCTTCTC
USP-WT-Salk_092647R	GCTCTTTCGCAGTCTTAATGTATG
Salk-LB	CGTGGACCGCTTGCTGCAACT
pMiox4-F	TAAGCTTCATAGTCGAGCAAGAAGTTCAA
pMiox4-R	TACTAGTCTTTTCGAAGAAAGGTTTTTAAAAGAG
pLAT52-F	TAAGCTTAATCAAATCAAAAAGAAAAATGA
pLAT52-R	TACTAGTTCTTTTATTAGAGATTTTTGTTTTGTTTT
pUbq10-F	TAAGCTTCTCTTATAAGTTACAAACC
pUbq10-R	TACTAGTCTTGATCACGGTAGAGAGAATTG

Suppl. Fig. S1 (Gene expression) The AtGenexpress microarray dataset was analyzed for expression of genes involved in the recycling pathway to nucleotide sugars. The maximum value for the organs root, rosette leaves, shoot, flower and stamen was set to 100% and is represented as a heat map by dark blue squares.

Suppl. Fig. S2

Separation and staining of AGPs. Total AGPs were separated on an agarose gel and stained with

Yariv. (1) wild type; (2) *ara1-1*; (3) *kd-usp*.

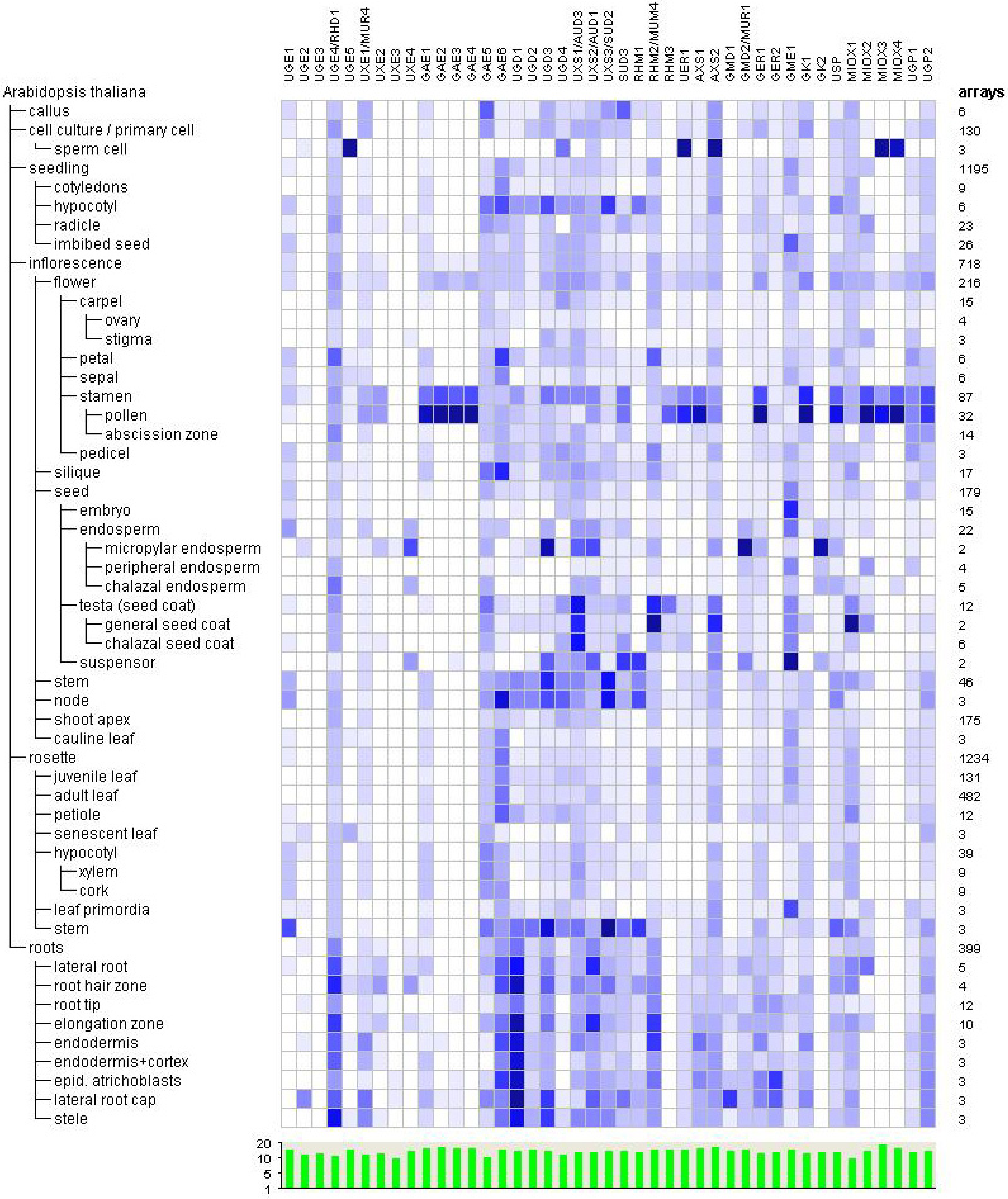

UGE1
UGE2
UGE3
UGE4/RHD1
UGE5
UXE1/MUR4
UXE2
UXE3
UXE4
GAE1
GAE2
GAE3
GAE4
GAE5
GAE6
UGD1
UGD2
UGD3
UGD4
UXS1/AUD3
UXS2/AUD1
UXS3/SUD2
SUD3
RHM1
RHM2/MUM4
RHM3
UER1
AXS1
AXS2
GMD1
GMD2/MUR1
GER1
GER2
GME1
GK1
GK2
USP
MIOX1
MIOX2
MIOX3
MIOX4
UGP1
UGP2
Arabidopsis thaliana
arrays
callus 6
cell culture / primary cell 130
sperm cell 3
seedling 1195
cotyledons 9
hypocotyl 6
radicle 23
imbibed seed 26
inflorescence 718
flower 216
carpel 15
ovary 4
stigma 3
petal 6
sepal 6
stamen 87
pollen 32
abscission zone 14
pedicel 3
silique 17
seed 179
embryo 15
endosperm 22
micropylar endosperm 2
peripheral endosperm 4
chalazal endosperm 5
testa (seed coat) 12
general seed coat 2
chalazal seed coat 6
suspensor 2
stem 46
node 3
shoot apex 175
cauline leaf 3
rosette 1234
juvenile leaf 131
adult leaf 482
petiole 12
senescent leaf 3
hypocotyl 39
xylem 9
cork 9
leaf primordia 3
stem 3
roots 399
lateral root 5
root hair zone 4
root tip 12
elongation zone 10
endodermis 3
endodermis+cortex 3
epid. atrichoblasts 3
lateral root cap 3
stele 3
20
10
5
1

Fig. S2

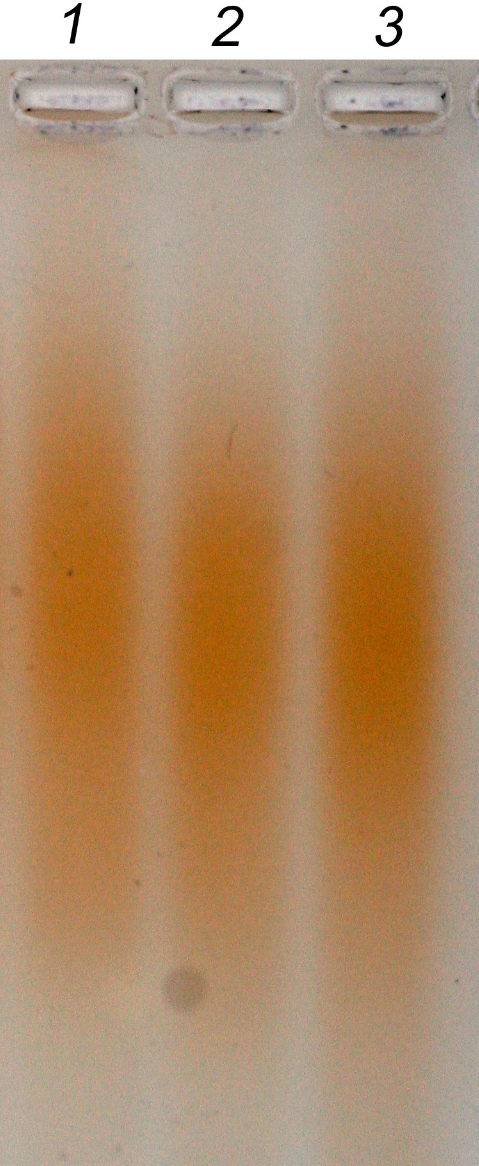

Supplement: Supplementary file 1 [file tpj0074-0239-SD1.pdf]
